# Zinc Deficiency and the Severity of Pneumonia in Vietnamese Children: A Hospital-Based Study

**DOI:** 10.7759/cureus.65771

**Published:** 2024-07-30

**Authors:** Qui Phu La, Son Hoang Le, Phuong Minh Nguyen, Ly Cong Tran

**Affiliations:** 1 Department of Pediatrics, Can Tho University of Medicine and Pharmacy, Can Tho City, VNM

**Keywords:** vietnam, children, prevalence, pneumonia, zinc deficiency

## Abstract

Background

Pneumonia is a critical global health concern that often results in severe complications and fatalities, especially among young children. Zinc plays a crucial role in immune function and maintaining respiratory epithelial integrity. Despite its importance, data on the prevalence of zinc deficiency and its impact on pneumonia severity in Vietnamese children are limited.

Objectives

This study aimed to investigate the prevalence of zinc deficiency and its association with pneumonia severity in Vietnamese children under five years old. The findings could significantly contribute to our understanding of the role of zinc in pneumonia severity, guiding future public health interventions, nutritional policies, and clinical practices to prevent zinc deficiency and reduce pneumonia morbidity and mortality in children.

Methods

An analytical cross-sectional study was conducted at a major pediatric center in Southwestern Vietnam from December 2022 to February 2024, involving 222 children aged 2 to 59 months diagnosed with pneumonia. Clinical assessments and laboratory measurements, including serum zinc levels, were performed. Statistical analyses were conducted to compare clinical characteristics and outcomes between zinc-deficient and non-deficient groups. Multivariable logistic regression was used to assess the association between zinc deficiency and pneumonia severity, with statistical significance set at p<0.05.

Results

The prevalence of zinc deficiency among children with pneumonia was 74.3%. Zinc-deficient children showed a significantly higher proportion of severe pneumonia (57.6% vs. 8.8%, p<0.001), as well as a higher proportion of high fever, poor feeding, vomiting, and respiratory distress compared to non-deficient children (p<0.001). Multivariable logistic regression identified zinc deficiency as an independent predictor of severe pneumonia (aOR=13.1, 95% CI: 4.7-36.8, p<0.001).

Conclusion

Zinc deficiency was prevalent among Vietnamese children with pneumonia and was associated with an increased risk of severe pneumonia.

## Introduction

Pneumonia remains a pervasive disease and a leading cause of mortality among children worldwide. Every 43 seconds, a child under the age of five years dies from pneumonia [1]. Zinc, an essential micronutrient involved in over 300 enzymatic reactions, plays a crucial role in immune function and maintaining the integrity of respiratory epithelial cells. Zinc deficiency compromises immune responses and increases susceptibility to infections, including pneumonia [2,3]. In low- to middle-income countries, the prevalence of zinc deficiency among children ranges from 23% to 82%, with about 50% of these countries reporting rates exceeding 50% [4]. In Vietnam, recent data from the Ministry of Health in 2021 indicates that 58% of children have zinc deficiency [5]. Recent studies conducted in Asian countries have yielded varying findings regarding the role of zinc in childhood pneumonia. Some investigations have found that lower serum zinc levels correlate with more severe pneumonia, prolonged hospitalization, worsened symptoms, and increased mortality [6-10]. However, the prevalence of zinc deficiency specifically in Vietnamese children under five years old with pneumonia has not been documented, highlighting a significant gap in understanding and addressing this critical health issue.

Therefore, this current study aimed to investigate the prevalence of zinc deficiency and its clinical and paraclinical characteristics among Vietnamese children with pneumonia. Additionally, it sought to determine whether zinc deficiency is associated with pneumonia severity in these children. Insights gained from this study could inform targeted interventions and policies aimed at reducing the burden of zinc deficiency-related pneumonia, thus fostering stronger healthcare systems and improving child health outcomes across Vietnam.

## Materials and methods

Study design and settings

This analytical cross-sectional study enrolled 222 children aged 2 to 59 months who were diagnosed with pneumonia and admitted to Can Tho Children's Hospital, the largest pediatric facility in the Southwestern region of Vietnam, from December 2022 to February 2024. Based on an anticipated prevalence of zinc deficiency among children with pneumonia of 72% [11], with a margin of error of 6% and a 95% confidence level, and a proposed attrition rate of 3%, the calculated sample size was determined to be 222 children. Participants were consecutively sampled until the required sample size was reached.

Children included in the study had been diagnosed with pneumonia by pediatricians at the time of admission. Children were excluded from the study if they had any of the following conditions: (1) congenital anomalies (such as congenital heart or lung disease), (2) inherited disorders of zinc metabolism (e.g., Acrodermatitis enteropathica), (3) chronic liver and kidney disease, (4) acute diarrhea at the time of serum zinc concentration testing or a history of acute diarrhea within the past month, (5) zinc supplementation within three months before hospitalization.

Data collection

A consistent assessment was conducted for every child included in the current study upon admission by the pediatrician investigator and recorded on a paper data collection form, including demographics (age, sex, location), medical history (previous pneumonia episodes, low birth weight, preterm birth), clinical signs and symptoms (time from symptom onset to hospitalization, temperature, feeding status, vomiting, cough, runny nose, wheezing, tachypnea, crackles, chest indrawing, nasal flaring, cyanosis, oxygen saturation (SpO2) levels) and relevant laboratory investigations such as serum zinc levels measurement, complete blood count analysis, and C-reactive protein quantification.

Pneumonia is characterized by cough and/or difficulty breathing, with or without fever, and at least one of the following signs [12,13]: (1) age-specific tachypnea (≥50 breaths per minute for ages 2 to <12 months, or ≥40 breaths per minute for ages 1 to 5 years); (2) chest indrawing (visible indrawing of the lower chest wall during inhalation); (3) abnormal lung auscultation findings (diminished breath sounds and/or adventitious breath sounds such as crackles, bronchial breath sounds, or crepitations).

Severe pneumonia is diagnosed when a child with pneumonia exhibits at least one of the following signs [14]: (1) respiratory rate >70 breaths per minute in infants or >50 breaths per minute in older children; (2) moderate to severe suprasternal, intercostal, or subcostal retractions; (3) grunting or nasal flaring; (4) intermittent apnea; (5) cyanosis; (6) inability to breastfeed (in infants) or signs of dehydration (in older children); (7) capillary refill time ≥2 seconds.

Zinc Assessment

Venous blood samples were collected from each child, and serum was separated. Serum zinc concentrations were measured using a colorimetric assay on an AU480 Chemistry Analyzer (Beckman Coulter, Inc., United States). The cut-off value for zinc deficiency was defined as <10.7 μmol/L, based on established research [15].

Children were considered undernourished if their weight-for-age, height-for-age, and weight-for-height Z-scores were below -2 SD [16]. Anemia was diagnosed if hemoglobin levels were below -2SD for age [17].

Ethical approval

This study adhered to the ethical standards of the institutional and national research committees, following the principles outlined in the 1964 Helsinki Declaration and its subsequent amendments or equivalent ethical standards. Ethical approval for the study was granted by the Ethics Committee in Biomedical Research at Can Tho University of Medicine and Pharmacy (No. 177/PCT-HĐĐĐ). Before participating in the study, informed consent was obtained from the parents or guardians of all participating children, who were assured of their right to withdraw their child from the study at any time without affecting the child's medical care. All children’s information was kept confidential.

Statistical analysis

All statistical analyses were conducted using IBM SPSS Statistics version 26.0 (IBM Corp., Armonk, New York, United States). Descriptive statistics were employed to summarize baseline characteristics of the study population, presenting categorical variables as frequencies and percentages. Categorical variables were compared using the Chi-square test or Fisher's exact test (if >20% of cells had expected counts <5). Continuous variables were assessed using the independent t-test for normally distributed data. Statistical significance was set at p <0.05.

To investigate the association between zinc deficiency and pneumonia severity, univariable and multivariable logistic regression analyses were employed. In addition to age and time from symptom onset to hospital admission, which could influence pneumonia severity, other potential risk factors were initially evaluated in univariable logistic regression models. Variables with p <0.05 were subsequently included in a multivariable logistic regression model to identify independent predictors of severe pneumonia.

## Results

A total of 222 children aged 2 to 59 months hospitalized with pneumonia at our center during the study period were included. Among all the children, 157 (74.3%) were found to have zinc deficiency, while 57 (25.7%) did not exhibit zinc deficiency. The proportion of male patients was 64%, which is 1.77 times higher than that of female patients. The majority of children with pneumonia were aged between 12 and 35 months. Further details on additional characteristics are presented in Table 1.

**Table 1:** General characteristics and the prevalence of zinc deficiency of the study participants.

Characteristics	Number of patients	Percentage
Sex		
Male	142	64%
Female	80	36%
Age group		
2-<12 months	65	29.3%
12-35 months	117	52.7%
36-59 months	40	18%
Location		
Urban	96	43.2%
Rural	126	56.8%
Nutritional status		
Undernutrition	46	20.7%
Overweight/Obesity	8	3.6%
Normal	168	75.7%
History of pneumonia		
≥ 2 episodes	18	8.1%
< 2 episodes	204	90.9%
Zinc deficiency		
Yes	165	74.3%
No	57	25.7%

The clinical and paraclinical characteristics of children with pneumonia are detailed in Table 2. Children with pneumonia and zinc deficiency exhibited a significantly higher prevalence of clinical symptoms, including high fever, digestive disorders (poor feeding, vomiting), tachypnea, and wheezing (p<0.001). Specifically, the occurrence of moderate to severe respiratory distress symptoms (chest indrawing, nasal flaring, SpO2 <94%) was significantly more frequent in the zinc-deficient group (p<0.001). Sixty-one (37%) of the zinc-deficient patients had anemia, which was significantly higher than in the non-zinc-deficient group, where 11 (19.3%) patients had anemia (p=0.014).

**Table 2:** Clinical and paraclinical characteristics and severity of pneumonia in children with and without zinc deficiency. ^a^Chi-Square test for independence, with ꭓ^2^-statistic (df) value;  ^b^Fisher’s Exact test; ^c^High fever: ≥39 degrees Celcius; MCV: Mean corpuscular volume; MCH: Mean corpuscular hemoglobin; CRP: C-reactive protein; SpO_2_: Saturation of peripheral oxygen.

Characteristics	Total, n (%)	Zinc deficiency		Statistical value	P-value
		Yes, n (%)	No, n (%)		
Clinical features					
High fever^c^	103 (46.4)	89 (53.9)	14 (24.5)	14.70 (1)	<0.001^a^
Poor feeding	77 (34.7)	74 (44.8)	3 (5.3)	29.30 (1)	<0.001^a^
Vomiting	126 (56.8)	111 (67.3)	15 (26.3)	28.96 (1)	<0.001^a^
Cough	222 (100)	165 (100)	57 (100)	-	1.000
Runny nose	152 (68.5)	117 (70.9)	35 (61.4)	1.77 (1)	0.183^a^
Wheezing	167 (75.2)	143 (86.7)	24 (42.1)	45.14 (1)	<0.001^a^
Tachypnea	205 (92.3)	162 (98.2)	43 (75.4)	-	<0.001^b^
Crackles	214 (96.4)	161 (97.6)	53 (93)	-	0.208^b^
Chest indrawing	169 (76.1)	140 (84.8)	29 (50.9)	26.90 (1)	<0.001^a^
Nasal flaring	26 (11.7)	26 (15.8)	0 (0)	10.17 (1)	0.001^a^
Cyanosis	22 (9.9)	20 (12.1)	2 (3.5)	3.52 (1)	0.061^a^
SpO_2_<94%	51 (23)	48 (29.1)	3 (5.3)	13.59 (1)	<0.001^a^
Laboratory features					
Anemia	72 (32.4)	61 (37)	11 (19.3)	6.04 (1)	0.014^a^
Low MCV and MCH	128 (57.7)	100 (60.6)	28 (49.1)	2.29 (1)	0.130^a^
Leukocytosis	77 (34.7)	57 (34.5)	20 (35.1)	0.005 (1)	0.941^a^
Elevated CRP	141 (66.5)	108 (68.4)	33 (61.1)	0.95 (1)	0.330^a^
Severity of pneumonia					
Severe pneumonia	100 (45)	95 (57.6)	5 (8.8)	40.76 (1)	<0.001^a^

Moreover, among children with zinc deficiency, 95 (57.6%) had severe pneumonia, whereas only 5 (8.8%) of those without zinc deficiency had severe pneumonia. Additionally, comparing the mean zinc concentrations between the pneumonia and severe pneumonia groups revealed significantly lower levels in children with severe pneumonia (7.74±2.1 µmol/L) compared to those with non-severe pneumonia (10.47±4.03 µmol/L) (p<0.001).

Based on the univariable logistic regression analysis presented in Table 3, zinc deficiency, undernutrition, anemia, a history of pneumonia two or more times, low birth weight, and preterm birth were all associated with an increased risk of severe pneumonia. Furthermore, following adjustment in a multivariable logistic regression model, zinc deficiency was independently associated with severe pneumonia, demonstrating an adjusted odds ratio of 13.1 (95% CI: 4.7-36.8; p<0.001).

**Table 3:** Univariable and multivariable logistic regression analyses of risk factors for severe pneumonia. ^†^Wald test; ^*^p-value <0.05 was considered significant; cOR: crude Odds Ratio; aOR: adjusted Odds Ratio; 95% CI: 95% confidence interval.

Factors	Univariable analysis			Multivariable analysis		
	cOR (95% CI)	Wald (df)	P-value^†^	aOR (95% CI)	Wald (df)	P-value^†^
Zinc deficiency	14.1 (5.4-37.2)	28.72 (1)	<0.001^*^	13.1 (4.7-36.8)	24.02 (1)	<0.001^*^
Undernutrition	2.3 (1.2-4.4)	5.71 (1)	0.017^*^	1.4 (0.65-3.1)	0.80 (1)	0.371
Anemia	2.4 (1.4-4.3)	9.07 (1)	0.003^*^	1.9 (0.97-3.6)	3.50 (1)	0.061
History of pneumonia ≥ two episodes	4.8 (1.5-15)	7.02 (1)	0.007^*^	4.3 (1.12-16.3)	4.52 (1)	0.034^*^
Low birth weight (<2500g)	2.8 (1.5-5.1)	10.72 (1)	0.001^*^	1.2 (0.54-2.9)	0.24 (1)	0.625
Preterm birth (<37 weeks)	2.1 (1.15-3.7)	5.82 (1)	0.016^*^	1.3 (0.52-3.1)	0.33 (1)	0.566
Age group (<12 months)	1.2 (0.65-2.1)	0.26 (1)	0.610	1.6 (0.77-3.2)	1.51 (1)	0.219
Time from symptom onset to hospitalization (days)	0.95 (0.88-1.02)	1.87 (1)	0.172	0.94 (0.87-1.02)	1.86 (1)	0.172

## Discussion

Our study observed a higher proportion of male children (64%) compared to female children (36%), resulting in a male-to-female ratio of 1.7:1. This finding is consistent with previous studies, such as that by Kumar A and Prakash J (62% male) [10]. The higher prevalence of pneumonia in male children may be attributed to differences in respiratory anatomy. Studies have shown that males are more likely to get pneumonia and experience greater severity when infected due to anatomical differences. Boys have smaller peripheral airways than girls of the same age before puberty, particularly those under one year old. This narrower airway diameter increases the risk of obstruction due to inflammation and secretions during lower respiratory tract infections, leading to higher hospitalization rates in males compared to females. Additionally, the role of estrogen (E2) in modulating inflammation and the more robust innate and adaptive immune responses observed in females compared to males [18,19] is highlighted. Children aged 12 to 35 months had the highest prevalence of pneumonia (52.7%). This observation aligns with findings from Rajasekaran J et al. [8] and Kumar N et al. [20], who also reported similar age distributions. This may be explained by waning maternal antibodies, immature respiratory systems, and increased environmental exposure in this age group [13]. As children age, their immune and respiratory systems mature, and they acquire antibodies through pathogen exposure, reducing the risk of pneumonia. Furthermore, older children may benefit from enhanced parental care and vaccination efforts.

The prevalence of zinc deficiency among children with pneumonia in our study was 74.3%, closely aligning with the 72% reported by Naushad AA et al. [11]. This rate exceeds the 62% documented by Rajasekaran J et al. [8], indicating that geographic location, cultural practices, and living conditions may influence zinc deficiency prevalence. Nevertheless, all studies consistently report a higher prevalence of zinc deficiency in children with pneumonia compared to the general population under five years old, as shown by Chu M et al. (28.6%) [21], and the Ministry of Health in 2021 (58%) [5]. This pattern is corroborated by case-control studies, such as Rajasekaran J et al. (62% zinc deficiency in the pneumonia group vs. 18% in the control group) [8], and Naushad AA et al. (72% vs. 27%, p<0.001) [11]. Furthermore, studies by Kumar A and Prakash J et al. [10], and Hamed MM et al. [9] also reported significantly lower serum zinc concentrations in children with pneumonia than controls (p<0.001). Thus, zinc deficiency remains notably prevalent in children with pneumonia. Addressing zinc deficiency and associated risk factors in the Vietnamese pediatric population is crucial, as zinc supplementation may reduce the incidence and prevalence of pneumonia.

In our study, 53.9% of children with zinc deficiency presented with high fever, compared to 24.5% of children without zinc deficiency (p<0.001). This finding aligns with the observations of Oguz S et al., who reported significantly lower serum zinc concentrations in children with fever but without seizures, compared to those without fever (p<0.05) [22]. Furthermore, Ahmed AF et al. demonstrated that zinc supplementation in children with pneumonia significantly reduced fever duration compared to placebo (p=0.001) [23]. These findings can be attributed to zinc's critical role in modulating inflammatory responses. Zinc deficiency may exacerbate inflammatory responses, leading to higher fever in infected children [2]. Although fever is a natural defense mechanism against infection, prolonged high fever can result in complications such as seizures, dehydration, and exhaustion.

Regarding GI symptoms, zinc-deficient children exhibited significantly higher rates of poor feeding (44.8%) and vomiting (67.3%) compared to non-zinc-deficient children (5.3% and 26.3%, respectively) (p<0.001). Zinc deficiency may contribute to these symptoms by reducing the activity of neuropeptide Y, an appetite stimulant, and impairing neurotransmitter function, potentially leading to refusal to feed. Additionally, zinc deficiency can decrease the activity of digestive enzymes and disrupt the intestinal epithelial barrier, potentially resulting in poor digestion, nausea, and vomiting [24]. These effects may be exacerbated in children with pneumonia, especially severe pneumonia, as gastrointestinal disturbances often occur during the disease's initial and acute stages [13]. Laghari GS et al. observed that 16% of children with poor feeding after 48 hours showed improvement with 20mg/day zinc supplementation as an adjunct to standard treatment, with a significant difference compared to the placebo group (p=0.01) [25]. Currently, there is a lack of research on gastrointestinal disorders in children with both pneumonia and zinc deficiency. Further studies are needed to explore the clinical characteristics of this population and to determine if there are distinct differences in gastrointestinal symptoms between zinc-deficient and non-zinc-deficient children with pneumonia.

Children with zinc deficiency exhibited a significantly higher rate of tachypnea compared to those without zinc deficiency (98.2% vs. 75.4%, p<0.001). This observation aligns with findings from Kumar N et al., who also reported an association between zinc deficiency and tachypnea in children with pneumonia [20]. Laghari GS et al. (2019) noted a reduction in tachypnea from 78% to 26% after 48 hours of 20 mg/day elemental zinc supplementation in addition to standard treatment, with a significant difference compared to the placebo group (p<0.001) [25]. These findings may be explained by zinc's role in modulating inflammation and maintaining respiratory epithelial integrity. Zinc deficiency can exacerbate the inflammatory response, leading to increased damage to alveoli and airways [2,3]. Furthermore, zinc deficiency may enhance mucus secretion in the respiratory tract of children with pneumonia [26], contributing to the high prevalence of tachypnea in this group.

Wheezing was significantly more prevalent in zinc-deficient children with pneumonia (86.7%) compared to non-zinc-deficient children (42.1%). Similarly, de Cássia Ribeiro-Silva R et al., in a study conducted in Brazil, found that children with low serum zinc concentrations had a 1.9-fold increased risk of wheezing compared to those with higher zinc levels [27]. Zinc deficiency can elevate oxidative stress, disrupt the Th1/Th2 balance, and increase the production of inflammatory cytokines [2]. This imbalance can contribute to enhanced mucus secretion and bronchoconstriction [26]. Zinc is essential for modulating inflammatory responses and reducing the production of inflammatory mediators such as leukotrienes and histamine, which are implicated in bronchoconstriction and mucus hypersecretion [2,26].

Our study identified a significantly higher prevalence of moderate to severe respiratory distress symptoms in zinc-deficient children with pneumonia, including chest retractions, nasal flaring, and SpO2 below 94%, compared to non-zinc-deficient children (p<0.001). These findings are consistent with those of Saleh NY et al., who observed a significant association between lower zinc levels and increased severity of respiratory distress [7]. Hamed MM et al. also reported a stepwise decrease in mean zinc concentrations correlating with the severity of respiratory distress (11.09±3.78 µmol/L in mild cases, 9.39±3.29 µmol/L in moderate cases, and 8.44±2.63 µmol/L in severe cases; p=0.002) [9]. Similarly, Agarwal A et al. noted a significantly higher prevalence of SpO2 below 94% in children with lower serum zinc levels (p<0.05) [28]. Although the rate of cyanosis was higher in the zinc-deficient group (12.1%) compared to the zinc-sufficient group (3.5%), this difference did not reach statistical significance (p=0.061).

These results suggest that zinc deficiency may increase the risk and severity of respiratory distress in children with pneumonia. Zinc deficiency may exacerbate respiratory distress through several mechanisms. It impairs immune cell function, leading to increased susceptibility to infections and a more severe inflammatory response [2,3]. Additionally, zinc deficiency can diminish respiratory muscle mass and strength by impairing protein synthesis and mitochondrial function, thereby compromising respiratory function [29].

We observed a significantly higher prevalence of anemia in children with pneumonia and zinc deficiency (37%) compared to those without zinc deficiency (19.3%) (p<0.05). This finding is consistent with the study by Hussain AM et al., which reported significantly lower serum zinc concentrations in anemic children compared to non-anemic children [6]. Naushad AA et al. also concluded that zinc deficiency is associated with anemia (p=0.007) [11]. This association may be explained by zinc's crucial role in heme synthesis and antioxidant defense. Zinc acts as a cofactor for enzymes such as carbonic anhydrase and delta-aminolevulinic acid dehydratase, which are essential for heme production, a critical component of hemoglobin. Zinc deficiency can inhibit these enzymes, leading to reduced hemoglobin synthesis, impaired RBC production, and subsequently, anemia. Additionally, zinc is involved in various protein systems that mitigate oxidative stress and repair damage to cellular macromolecules. Consequently, zinc deficiency increases the susceptibility of RBCs to free radicals, leading to increased hemolysis and anemia [30].

Our analysis revealed a considerably higher proportion of severe pneumonia cases among zinc-deficient children (57.6%) than those with sufficient zinc levels (8.8%). Furthermore, we observed significantly lower mean zinc concentrations in children with severe pneumonia (7.74±2.1 µmol/L) compared to those with non-severe pneumonia (10.47±4.03 µmol/L) (p<0.001). Studies from other countries by Saleh NY et al. [7], Kumar A and Prakash J [10], Hamed MM et al. [9], and Agarwal A and Hasan S [28] consistently reported lower serum zinc concentrations in children with severe pneumonia compared to those with non-severe pneumonia (p<0.001). In our study, we sought to determine whether zinc deficiency contributes to an increased risk of severe pneumonia in children already afflicted with pneumonia. Utilizing a multivariable logistic regression model adjusted for potential confounding factors that could impact pneumonia severity, our analysis identified two remaining significant factors. Zinc deficiency emerged as the most influential predictor, with an adjusted odds ratio (aOR) of 13.1 (95% CI: 4.7-36.8; p<0.001). This indicates that zinc deficiency markedly increases the odds of severe pneumonia by a striking 13-fold compared to adequate zinc levels. According to a previous study by Kumar N et al. in India, zinc-deficient children had an odds ratio of 16 for severe pneumonia compared to those without zinc deficiency (95% CI: 6.01-42.63), which supports our results [20]. As discussed above, zinc deficiency exacerbates inflammatory responses, impairs mucus clearance, compromises immune function against infection, increases the risk of moderate to severe respiratory distress, and causes more frequent gastrointestinal problems (vomiting, poor feeding), leading to reduced energy intake in children with pneumonia. Therefore, zinc deficiency may increase the risk of developing severe pneumonia compared to zinc sufficiency. Consequently, preventing and treating zinc deficiency could reduce the burden of severe pneumonia.

Although this study provides valuable insights, it has several limitations. First, it was conducted at a single center, which may limit the generalizability of the findings to the broader population of children with pneumonia. Second, the absence of a control group prevents us from comparing the prevalence of zinc deficiency in children with pneumonia to that in the general population. Additionally, the cross-sectional design can identify associations but cannot establish causality. Lastly, the lack of follow-up precludes us from determining the long-term impact of altered serum zinc levels in children with pneumonia. To enhance the generalizability and validity of these findings, future research should expand to multiple centers across Vietnam. A longitudinal component should be incorporated to assess the long-term impact of zinc deficiency on pneumonia outcomes. Furthermore, studies should explore other potential risk factors for zinc deficiency and examine the cost-effectiveness of interventions to address zinc deficiency in Vietnam. Future research should also investigate the efficacy of zinc supplementation in preventing and reducing the severity of pneumonia through randomized controlled trials.

## Conclusions

In conclusion, our study reveals a substantial prevalence of zinc deficiency (74.3%) among children aged 2 to 59 months with pneumonia, which is considerably higher than the national average. Zinc-deficient children exhibited a higher prevalence of symptoms including high fever, vomiting, poor feeding, tachypnea, wheezing, and signs of moderate to severe respiratory distress compared to their non-zinc-deficient counterparts. Importantly, zinc deficiency independently conferred a 13-fold increased risk of developing severe pneumonia. These findings underscore the critical role of zinc status in influencing the severity of pneumonia and suggest the potential benefits of addressing zinc deficiency in pediatric pneumonia management strategies.
